# Evaluation of the regional nurse-supported hepatitis C shared care program in Western Australia: a mixed methods study

**DOI:** 10.1186/s12913-015-1055-1

**Published:** 2015-09-21

**Authors:** Roanna Lobo, Lester Mascarenhas, David Worthington, Judith Bevan, Donna B. Mak

**Affiliations:** Collaboration for Evidence, Research and Impact in Public Health, Curtin University, Bentley, WA 6845 Western Australia; Formerly Sexual Health and Blood-borne Virus Program, Communicable Disease Control Directorate, Western Australian Department of Health, Perth, Western Australia; Sexual Health and Blood-borne Virus Program, Communicable Disease Control Directorate, Western Australian Department of Health, Perth, Western Australia; School of Medicine, University of Notre Dame, Fremantle, Western Australia

## Abstract

**Background:**

Nurse-supported shared care services for patients living with hepatitis C have been implemented in some regional areas of Western Australia to provide access to local treatment and care services for patients and to improve currently low levels of treatment uptake. This study collected data from health professionals involved in managing the care of patients living with hepatitis C and from patients engaged in regional nurse-supported hepatitis C shared care services in Western Australia.

**Methods:**

Key informant qualitative interviews were conducted with health professionals in regions operating a nurse-supported shared care service and in regions without this service. Patients engaged in the shared care program at the time of the study were invited to complete a short questionnaire.

**Results:**

Nurse-supported shared care services reduced patient transport costs to tertiary centres, accelerated access to treatment and delivered >98 % compliance with treatment schedules. Patients engaged with regional hepatitis C shared care services expressed high levels of satisfaction and indicated that they would delay treatment if it was not available locally. Telehealth support from tertiary liver clinics and allied health services were available to health professionals engaged in regional shared care services and were used effectively. There was limited participation by general practitioners in regional hepatitis C shared care services and regional patients’ access to treatment was influenced by the availability and capacity of health professionals. Uptake of treatment and engagement in the regional shared care program was limited for Aboriginal people and younger people although these groups had the highest rates of hepatitis C notifications in Western Australia.

**Discussion:**

The patients consulted for this study preferred to access hepatitis C treatment and care locally rather than travel to tertiary liver clinics, up to 1500 kilometres away. The reasons for limited engagement in the shared care program by some groups with high rates of hepatitis C notifications requires further investigation. Health professionals identified several benefits of the shared care program including continuity of care for patients, shorter waiting times, longer appointment times and high levels of treatment compliance.

**Conclusions:**

Hepatitis nurses in regional areas can coordinate effective patient treatment and care when supported by treatment protocols and access to physicians and liver specialists, including through telehealth. Treatment and care options to suit individual preferences are required to avoid further stigmatising marginalised groups. The role of primary care in facilitating hepatitis C treatment uptake should be explored further including strategies for improving the participation of general practitioners in regional shared care services.

## Background

Hepatitis C is a viral infection of the liver and a major public health issue in Western Australia (WA) affecting around 0.8 % of the population with over 1,000 cases notified to the WA Department of Health each year [1]. The majority of notifications are in people aged 20 to 34 years and the most commonly reported risk factor for infection is injecting drug use [2]. In 2012, the WA newly acquired hepatitis C infection rate, where there is evidence of infection occurring in the last 24 months, was more than double the national rate, 5.3 compared with 2.6/100,000 population. For unspecified hepatitis C, where infections have unknown duration, notification rates in WA (43/100,000 population) remained comparable to national rates. Aboriginal^1^ to non-Aboriginal rate ratios for newly acquired and unspecified infections were higher, 13:1 and 7:1 respectively [2]. Of those infected, 75-80 % will develop chronic hepatitis C virus (HCV) infection and without treatment, 10-15 % of these patients will develop liver cirrhosis leading to liver cancer in approximately 5 % of patients [1].

Currently available treatments involving six- to nine- month courses of pegylated interferon alpha in combination with ribavirin or another oral antiviral drug offer a cure rate of around 60 % [3]. This rate is likely to increase to above 90 % with new oral treatments becoming available [4]. However, the number of people accessing hepatitis C treatment in Australia is low. There are an estimated 2,808 people receiving treatment through the Highly Specialised Drugs Program [5], equating to approximately 1.2 % of the estimated 230,000 people living in Australia with chronic hepatitis C infection [5]. Factors contributing to low uptake of treatment are varied and include lengthy treatment regimens, severe side effects, stigma and social inclusion issues, and limited availability of services [4, 6]. Diagnosis at a time prior to treatment being available, reluctance to begin treatment and lack of awareness on the part of both patients and general practitioners (GPs) about improvements in the available treatments may also be contributing factors [7].

The newer hepatitis C treatments which have shorter treatment schedules and fewer side effects may contribute to increased treatment uptake in Australia if they can be made available at an affordable cost. Stigma and social inclusion issues affecting treatment uptake have been addressed through various strategies. For example, a trial in New South Wales co-locating hepatitis C treatment services with other services for people who inject drugs (PWID) has shown promise in reducing the negative and stigmatising experiences reported by hepatitis C patients seeking treatment in other settings [8]. Hepatitis C treatment delivered through primary care settings has also been piloted successfully and may be an option for selected patients with HCV infection to improve access to treatment [6].

Shared care models also have a role in improving service accessibility for patients living with HCV infection. In the 2^nd^ edition of the Hepatitis C Resource Manual, shared care is described as “*a system that operates between GPs/physicians in rural or remote areas and liver specialists in major regional centres. The aim of shared care is to provide optimum input for people who live in rural or remote areas by reducing their travel time and expenses, whilst still having access to medical interventions*.” ([9], p.144). Multi-sectoral participation and partnerships between primary care providers and tertiary specialists are features of all shared care models.

Shared care models for hepatitis C have been trialled in Australia in the primary care sector with promising results [6, 7, 10, 11]. The benefits of shared care included improved service accessibility, increased patient compliance, increased likelihood of patients completing treatment, reduced travel costs for patients able to access treatment locally, and reduced demand on tertiary clinics for appointments [10, 11].

Given the high rates of HCV infection amongst PWID and high rates of imprisonment of PWID, custodial settings also present an opportunity to engage these marginalised populations. Shared care models targeting people in custodial settings have been shown to be effective in reducing the burden of HCV infection and increasing treatment uptake [12, 13].

Nurse-led models of shared care have been implemented for patients with chronic diseases in regional, rural and remote areas and have demonstrated additional benefits. Examples include: cultural acceptability for Aboriginal patients with renal disease [14]; helping to address the growing demand for chronic disease management by general practitioners [15]; and improved diabetes self-management by patients through opportunities to discuss complex issues with nurses more fully [16].

### WA regional nurse-supported hepatitis C shared care services

Regional nurse-supported hepatitis C shared care services were first implemented in the Great Southern and South West regions of WA in 2003 and have been available in the Kimberley region of WA since 2004. The services are funded by the WA Department of Health Sexual Health and Blood-borne Virus Program (SHBBVP) and employ dedicated hepatitis nurses to improve access to and uptake of hepatitis C treatment for patients with HCV living in regional, rural and remote areas.

The regional nurse-supported shared care program is a patient-centred model, primarily intended to deliver benefits to patients. Secondary benefits for health professionals may include support to engage patients in treatment and care and improved pathology workup and patient education prior to seeing the physician. The hepatitis nurses facilitate liaison between a patient and their GP, physician, and/or tertiary health services and assist patient access to allied health services including mental health services and drug and alcohol services. In 2011, WA had 183.5 GPs per 100,000 persons, the lowest per capita rate of GPs in Australia. The number of GPs in regional areas of Australia was also almost 57 % lower than in major cities [17].

This study evaluated the WA regional nurse-supported hepatitis C shared care program and the implications for WA regions without nurse-supported hepatitis C shared care services. The study collected data on the benefits, critical features, challenges and enablers of the shared care model and identified opportunities for improving access to hepatitis treatment and care for patients living in regional areas.

## Methods

WA health professionals involved in managing the care of patients with HCV infection (GPs, liver specialists, physicians, public health nurses and hepatitis nurses) and patients engaged with the WA regional nurse-supported hepatitis C shared care program provided data for this study.

### Key informant interviews

Sixteen health professionals involved in management of patients with hepatitis C were identified as key informants by the WA Department of Health SHBBVP. They received an email from the evaluation team informing them of the evaluation project and its aims and requesting their participation. In-depth semi-structured qualitative interviews were chosen for their potential to elicit rich information from the perspectives of key informants [18]. The interview questions were derived from interview guides used in a previous evaluation of hepatitis C shared care services. The questions explored which aspects of the program worked well and areas noted for improvement [11]. All interviews were conducted by a public health registrar (LM) who had no prior relationship with the key informants and who was independent of the WA nurse-supported regional shared care services program. In regions without a nurse-supported shared care service, semi-structured interviews were conducted by LM with physicians and public health nurses to understand any regional differences in services and patient needs. The interviews also aimed to assess the consequences and implications of the absence of a nurse-supported shared care program, including influences on service delivery, health service resources, and patients’ access to and uptake of treatment.

Interviews were conducted face-to-face whenever possible or by using web-cam based software or telephone. Written consent from participants was obtained to record the interviews to improve reliability of the data. Participation was voluntary and participants could withdraw from the study at any time.

The interview data were transcribed verbatim and thematic content analysis was used to establish an understanding of their meaning. The analysis process involved reading the transcripts and searching for patterns (“themes”) in the data which were then used to organise and describe the data, a process known as coding [19]. All transcripts were coded by another researcher (RL) to ensure that all themes had been identified and that coding was consistent. Confirmability of the interview transcripts and coding process was further supported through handwritten notes recorded by the interviewer (LM) [20].

### Patient survey

A short survey was created based on a patient survey used in a previous evaluation of hepatitis C shared care services [11]. The paper-based survey comprised 19 questions and included Yes/No questions, multiple choice questions and one open ended question. The survey was designed to be self-completed within 5–10 min and survey responses were anonymous. Written consent was required from participants. Fifty-one patients, comprising those currently receiving treatment or post-treatment follow-up, were engaged with the WA regional nurse-supported hepatitis C shared care program at the time of data collection. Regional hepatitis nurses posted the survey with an information sheet, consent form and reply paid envelope to 47 patients. Four patients were not invited to complete a survey; the reasons for this were not recorded.

The survey was anonymous and sought information on patient demographics; patterns of accessing health services; reasons for commencing treatment; the types of professionals primarily responsible for treatment management and support; perceptions of the quality of care, including access to medication, support and information; and overall impressions of and attitudes towards the shared care initiative. All patients received a reminder from their hepatitis nurse to complete the survey. The results of the patient survey were used to assess overall levels of patient satisfaction with the WA regional nurse-supported hepatitis C shared care program and to compare the data collected about patients’ experiences of treatment and care with data collected from health professionals involved in the shared care program.

Ethics approval for this study was obtained from the WA Country Health Service Human Research Ethics Committee (Ref: 2013:09). A reciprocal ethics agreement was also obtained from the Curtin University Human Research Ethics Committee (Approval Number HR11/2014).

## Results

The results of the key informant interviews and patient survey are presented below.

### Key informant interviews

Two key informants declined to participate, two did not respond to the invitation and one key informant was interested in the study but unable to find time to participate. Overall, 11 key informants were interviewed. These included the three regional hepatitis nurses, one GP and two physicians based in the regions, and a tertiary liver clinic specialist. Four key informant interviews were also held with physicians and public health nurses in regions which did not operate a nurse-supported hepatitis C shared care program. The interviews ranged in duration between 20 min and 90 min. The face-to-face and web-cam interviews were longer than the telephone interviews and enabled greater rapport between the interviewer and the key informants.

#### Current operation of the WA regional nurse-supported hepatitis C shared care model

The steps in the care pathway for a patient enrolled in the WA regional nurse-supported hepatitis C shared care program were mapped based on key informants’ answers. The steps are summarised below.

1. Self-referral by patient (or patient is referred by a GP) to the hepatitis nurse. The hepatitis nurse briefs the patient about the risks, benefits and precautions of treatment. The hepatitis nurse has several consultations with the patient, performing a thorough assessment and completing pathology workup before treatment can commence. For initial appointments, the hepatitis nurse would generally see patients face-to-face. Once treatment was commenced, some follow up may have been conducted over the telephone due to the distance that may otherwise have been required to travel (even within regions) to attend appointments.
2. The hepatitis nurse refers the patient to allied health services for psychological review or drug and alcohol assessment and support if needed. Access to allied health services in regional areas is variable and, depending on needs, may be provided through specialised services or managed by the GP, physician, or nurse. After tests are complete, the hepatitis nurse contacts the referring GP and makes recommendations to them about the patient’s condition and treatment.
3. When the patient consents to treatment the hepatitis nurse schedules an appointment for the patient to see the physician.
4. The physician liaises with the hepatitis nurse and the patient visits the nurse to receive their scripts, receive counselling and begin treatment.
5. The hepatitis nurse coordinates all patient treatment and care in consultation with the physician. The tertiary liver clinics provide advice and support to the physician and the hepatitis nurse on request.
6. Complex cases are referred to the tertiary liver clinics by the physician.

#### Access to treatment

The key informant interviews identified a range of factors that influenced whether regional patients received treatment and care when they needed it. Patient-related factors included: patient’s medical condition (e.g. existence of any co-morbidities); patient’s hepatitis C genotype (since treatment regimens can vary according to genotype); personal circumstances of patient (e.g. lifestyle, age and work commitments); patient’s choice given possible side effects; and patient’s willingness and commitment to attend initial appointments and complete the treatment schedule.

Other factors influencing whether patients received treatment were associated with the capacity of regions to treat and care for patients and included: delays in getting results from psychological and drug and alcohol investigations; capacity to follow up patients by specialist physicians; and the experience and capacity of the hepatitis nurse.

#### Roles of health professionals in shared care

The nurse was seen to provide a constant point of contact in a context of changing general practitioner locums in some regional areas.

*‘Most of the patients value the support from the nurse, she’s very conscientious and makes sure that they’ve had all their tests done and they’re up to date and they know what’s going on and she informs them what their test results are’ (Physician)*

*‘Patients don't want to see somebody different every time’ (Nurse)*

Tertiary centres provided education for GPs, nurses and physicians and advised on complex cases. Patients with cirrhosis or those requiring a liver transplant were referred to tertiary centres by physicians. Mental health and alcohol and drug services were generally available and patients could be referred to these services easily.

#### Perceived benefits of shared care services

Hepatitis nurses and physicians in the three regions with a nurse-supported shared care model reported a range of benefits including shorter waiting times; longer appointments; ability to be more responsive to patient needs; reduced transport costs for patients from regional locations to tertiary centres; increased adherence to treatment; and continuity of care.

*‘6 or 8 weeks to see a physician and then starting treatment within a week or two if they want to’. (Physician)*

*‘GP appointments are only 15 odd minutes. Most of the time when we [shared care service] see clients it’s 30 min. So we have time, we try to engage them quite holistically reviewing where they’re at with not just their physical health but other issues in their life’. (Nurse)*

In particular, patient compliance with the treatment schedule and the likelihood of patients completing treatment were very high, as indicated through compliance rates of 98 – 100 %.

*‘Very high probability patients will complete treatment. I can only think of one who stopped treatment’. (Nurse)*

#### Challenges and enablers associated with implementation

Challenges identified by key informants related to implementing a regional nurse-supported shared care model included: managing patients with high needs; high GP and medical registrar turnover in regional areas; and limited capacity of nurses and physicians to meet demands.

*‘We’ve got 6 patients waiting who we would be treating now if we had the nurse more days per week’. (Physician)*

Having an experienced nurse to decide when to consult a physician, dedicated time for nurses to discuss patients with a physician, and effective communication were noted as enablers for successful implementation of the shared care model.

*‘It has to be a fairly experienced nurse. You don’t just delegate it to someone without that clinical skill’. (Physician)*

*‘What works really well is the really good communication between the physicians and the nurse and the GP. So we’re really quite proactive about chasing people and communicating with each other exactly what we’re doing and what needs to be done’. (GP)*

Telehealth (videoconferencing) was used extensively. The majority of key informants who had used telehealth spoke positively about telehealth as an enabler to providing patient care and treatment.

*‘I think telehealth has got a role and I think it would be good to expand’. (Physician)*

Table 1 summarises the features identified by key informants as critical to the success of the WA regional nurse-supported hepatitis C shared care model.

**Table 1:** Critical features of the WA regional nurse-supported hepatitis C shared care model

Critical features of the WA regional nurse-supported hepatitis C shared care model
*Patient-centred care* enabling patients to choose when to start treatment depending on health status and personal or work commitments
*Dedicated hepatitis nurse located regionally* who is responsible for patient education and coordinating all patient treatment and care including follow up and monitoring of patients and patient referrals to allied health services
*Patient-centred care* enabling patients to choose when to start treatment depending on health status and personal or work commitments
*Specialist physicians* responsible for assessing patient’s health status, initiating treatment, and refining treatment schedule as required
*Collaboration with GPs* where possible to help patients manage side effects of treatment once initiated
*Telehealth links* between regional areas and tertiary liver clinics in Perth for ongoing support and for referral of complex cases according to agreed protocols
*Excellent communications* between nurse, GP, physician, and tertiary centres
*Continuity of care through* stability of key roles

#### Model of care in regions without a nurse-supported hepatitis C shared care service

In regions without a nurse-supported hepatitis C shared care program, both GP-driven and specialist-driven care pathways for patients were evident and telehealth was used.

*‘The liver clinic offers regions without a hepatitis C nurse surrogate nurse support by telehealth. We did a survey on telehealth and the patients were happy, although they worry about confidentiality’. (Physician)*

Patients were referred by GPs to a physician, if available, or to tertiary centres.

*‘The proportion of people that travel out of the region for treatment is zero. People are either treated within the region or they’re not treated because there’s no capacity’. (Physician)*

However, there appeared to be little awareness of what happened to patients once they were referred. The lack of local treatment follow-up for patients had implications for local emergency departments since patients with side effects from treatment may present to local emergency departments. A lack of GP involvement was also noted.

*‘Some GPs would like to get involved* [in shared care] *and some don’t. I think you’ve got to nurture those that do’. (Nurse)*

### Patient survey

Of the 47 patients invited to complete a survey, 22 (47 %) returned a completed survey. Two (2) surveys did not meet the requirements for the study and were excluded from analysis. Very few comments were received. Respondents ranged from 40 to 65 years of age. Table 2 shows the hepatitis C notification data (2010–2012) for the three regions currently implementing regional nurse-supported hepatitis C shared care services in Western Australia and outlines the basic demographic characteristics of patients who returned a survey.

**Table 2:** Demographic characteristics of hepatitis C notifications (2010–2012) [2], patients engaged in regional nurse supported shared care and survey respondents (June 2013) in the Kimberley, Great Southern and South West regions of Western Australia^a^

	Notifications (Newly acquired and unspecified 2010 – 2012) N (%)	Patients engaged in regional nurse-supported shared care program N (%)	Survey Respondents N (%)
Region			
Kimberley	72 (16.9 %)	13 (25 %)	2 (10 %)
Great Southern	109 (25.6 %)	16 (31 %)	4 (20 %)
South West	244 (57.4 %)	22 (44 %)	14 (70 %)
Gender			
Male	302 (71 %)	38 (74 %)	13 (65 %)
Female	123 (28.9 %)	13 (25 %)	7 (35 %)
Age group (yrs)			
≤49 years	328 (77 %)	23 (45 %)	5 (25 %)
≥50	97 (22.9 %)	28 (55 %)	15 (75 %)
Aboriginal			
Yes	74 (17.5 %)	2^b^ (4 %)	0 (0 %)
No	338 (79.5 %)	49 (96 %)	20 (100 %)
Unknown	13 (3.0 %)	0	0
Total	425	51	20 (100 %)

^a^Column totals may not summate to the total due to missing data

^b^No Aboriginal patients were receiving treatment at the time of the study, although two Aboriginal patients were still engaged in the shared-care program post-treatment

### Patient experiences of referral and treatment

The interval between referral and starting treatment ranged from less than one month to over two years, with a quarter of patients (25 %) reporting that they started treatment three to six months between referral and treatment.

Most patients identified the local ‘hepatitis C nurse’ or the ‘hepatitis C nurse and other’ as the main person involved in explaining their treatment (80 %), scheduling their appointments (60 %) and communicating their blood test results (90 %). The majority of patients (90 %) also reported it being ‘very easy’ or ‘easy’ to contact the hepatitis C nurse (Table 3).

**Table 3:** The main person identified by patients as being involved in explaining treatment, scheduling appointments and communicating blood test results^a^

	Hepatitis C nurse	Local specialist	Hepatitis C nurse and other	General Practitioners
Explaining treatment	12 (60 %)	3 (15 %)	4 (20 %)^b^	1 (5 %)
Scheduling appointments	10 (50 %)	7 (35 %)	2 (10 %)^b^	0
Communicating blood test results	14 (75 %)	2 (10 %)	3 (15 %)^c^	0

^a^Row totals may not summate to the total due to missing data

^b^‘Other’ included GP and specialist in local area

^c^‘Other’ included specialist in local area and PathWest pathology services

### Patient satisfaction

Patients reported high levels of satisfaction across the following three aspects of care: ‘Information received about the side-effects of treatment’, ‘Level of support received while on treatment’, ‘Overall experience of the hepatitis C treatment program’ (Table 4).

**Table 4:** Patient satisfaction levels with differing aspects of care

Number of respondents *n* = 20		Aspect of care		
		Information received about the side-effects of treatment	Level of support received while on treatment	Overall experience of the hepatitis C treatment program
Satisfaction level	Very satisfied	12 (60 %)	13 (65 %)	13 (65 %)
	Slightly satisfied	5 (25 %)	5 (25 %)	4 (20 %)
	Slightly unsatisfied	2 (10 %)	2 (10 %)	1 (5 %)
	Very unsatisfied	1 (5 %)	0	2 (10 %)
	Total	20 (100 %)	20 (100 %)	20 (100 %)

Eighty-five per cent of respondents were either very satisfied (65 %) or slightly satisfied (20 %) with the way the shared care program functioned in their area. Similarly, the majority of patients (90 %) were very satisfied (65 %) or slightly satisfied (25 %) with the level of support received while on treatment. Patients who reported lower levels of satisfaction requested more information on treatment side effects and the process from referral to treatment.

Having a dedicated hepatitis nurse who was able to facilitate access to local treatment was identified as a critical feature of the program. When asked what the favoured option would be if hepatitis C treatment was not available in their region, 12 (60 %) patients reported that they would wait longer until treatment became available in the region, three (15 %) patients reported that they would choose to see a private specialist in their region and a further three (15 %) patients indicated that they would travel to Perth for treatment. Data were missing for one patient and one patient was unsure.

#### Patient recommendations

Patients recommended that appointments with the regional hepatitis nurse and liver specialist should be maintained regularly throughout treatment, as they were not only considered to be important but were also reassuring for patients. One patient recommended that there should be staff members available to temporarily fill the positions of hepatitis nurse/specialists when they are on leave, which was identified as a limitation of nurse supported shared care in WA. The patient commented:

*‘The only problem I had during treatment was when my hepatitis nurse and my specialist went on holidays at the same time. They need someone to fill in for them’*.

Communication between patients, nurses and specialists was paramount to the overall satisfaction with the program and patients recommended nurses and specialists to maintain regular contact with patients, especially regarding treatment progression and side effects throughout treatment.

Travel/distance was mentioned by many patients, with one patient commenting on the length of time it took for a travel claim to be accepted.^2^ Another patient indicated that travelling a shorter distance to access medication would better suit their needs. There were no further patient comments about accessing medication although it is understood that medication is usually dispensed from the regional hospital pharmacy.

Two patients mentioned the blood test result process in their responses. One respondent would rather blood test results were sent directly to them and the other respondent would like to receive blood test results more regularly. The same respondent reported that they would have benefited from being linked to a support group during treatment.

## Discussion

This study evaluated the regional nurse-supported model of hepatitis C shared care in Western Australia. The findings indicated benefits for patients and health professionals including improved patient compliance and completion of treatment and reduced patient transport costs to tertiary centres. The study findings were consistent with those of other studies which identify a role for nurse-led models of care in regional areas to improve service accessibility [14–16] and for hepatitis C shared care models in the assessment and treatment of patients with HCV infection, particularly marginalised populations such as PWID and people in custodial settings [13, 21].

The waiting time to start treatment and the support services available to patients undergoing hepatitis C treatment in regions with a nurse-supported shared care hepatitis C program, seemed to be comparable, if not better, than in tertiary centres.

However, there was a limit to the number of patients that a hepatitis nurse and physician could support (about five to six patients per day that the nurse is employed). Nurse resource allocations should therefore be considered based on patient caseload in regional areas. This includes patients currently on treatment and those patients who require support post-treatment.

Participation of GPs in shared care services was considered to improve regional capacity to offer treatment to patients. However, there were very few incentives for GPs to be involved and GPs in regional areas were in short supply [17]. Other studies have suggested a minimum HCV patient caseload for GPs given the sometimes lengthy hiatus between diagnosis and commencing treatment for some HCV patients which can result in a lack of GP confidence and expertise if these skills are not required often [7]. In our study, a scarcity of GPs in regional areas, the perceived characteristics of hepatitis C patients as being non-compliant with medication or inconsistent in attending appointments, and the complexity of psychosocial issues often experienced by hepatitis C patients presented challenges to starting treatment.

The majority of patients expressed high levels of satisfaction with the services available and patients preferred to access treatment locally. However, since there is currently a much wider cohort of people who are *not* accessing treatment, it is not possible to generalise based on the patient data collected. For example, the results suggested that there may be barriers to treatment uptake and engagement of Aboriginal patients and younger patients in the shared care program. Aboriginal people comprised approximately 21 % of hepatitis C notifications in 2012 in the WA regions operating a shared care program. However, at the time of this study no Aboriginal patients were receiving treatment through the shared care program (although two Aboriginal patients were later identified as receiving post-treatment follow-up) [2]. Similarly, in 2012, notifications for newly acquired and unspecified hepatitis C were highest in both the 20 to 29 years and 50 years and over age groups respectively [2] while most patients engaged in the shared care programs were aged 50 years or older. The reasons for these discrepancies are unclear and further investigation of contributing factors is required. This may include understanding where Aboriginal people and younger people access testing and receive their diagnosis, how long patients wait before starting treatment, the reasons for delaying treatment, assessment of culturally safe health services, and the availability of allied health services.

In our study, health professionals perceived that the support of dedicated hepatitis C nurses helped to speed up access to treatment and enabled high levels of treatment compliance (>98 %). Telehealth was considered highly effective and a viable option when supported by appropriately trained health professionals and clear treatment protocols. This finding was consistent with another WA study [22] and the findings of Project ECHO (Extension for Community Healthcare Outcomes) which found treatment of HCV patients at 21 rural ECHO sites and prisons in New Mexico using telehealth to be comparable to treatment of patients at an HCV clinic in reaching a sustained virological response [21].

For regions that did not have a hepatitis nurse, the barriers to a patient starting treatment were significant and the likelihood was that patients would be unable to start treatment unless they had a long-term GP and/or were able to travel back and forth to tertiary liver services in Perth (over 1500 km from some regional areas). Not having nurse-led programs was perceived to be detrimental to people living with hepatitis C in regional areas. The limited GP and physician capacity in these areas meant that effective treatment could not be provided without nurse support.

The decision to implement regional shared care services needs to be considered carefully. For example, feasibility of regional hepatitis C shared care models requires the availability of allied health services such as mental health and drug and alcohol services in regional areas. In addition, people living with hepatitis C can experience stigma and discrimination and some patients in regional communities may prefer to travel to tertiary centres for reasons of anonymity. Treatment and care options to suit individual preferences, including for those in hard to service geographical areas, are therefore needed.

### Methodological considerations

The purpose of this study was to evaluate the nurse-supported hepatitis C shared care services in regional WA. Three limitations of the study should be noted. First, we were unable to follow up those patients who did not return a survey following a reminder from their nurse. The majority of surveys were completed in the South West region of WA and therefore the results of the patient survey were most representative of this region. Second, data were collected from patients who were *currently engaged* in the shared care program. The experiences of patients who were considering (or had declined) engaging in shared care services were not possible to collect. Finally, the participation of GPs in shared care services was considered important by all key informants. However, and perhaps related to GP capacity or motivation for participation in shared care, we were only able to recruit one GP for this study. The reasons for limited GP participation and strategies for increasing GP participation in regional shared care services should be explored further. GP involvement in hepatitis C shared care services may become more relevant when oral-only hepatitis C treatments become available, and if these are able to be prescribed by GPs.

## Conclusions

Hepatitis nurses in regional areas can coordinate effective patient treatment and care when supported by treatment protocols and access to physicians and liver specialists as needed. Telehealth was highly effective in regional areas and a viable option when supported by appropriately trained health professionals in the region. Our findings indicated that the nurse-supported model reduced patient transport costs to tertiary centres, accelerated access to treatment and delivered high levels of treatment compliance (>98 %). Furthermore, the patients consulted in our study preferred to access treatment locally and indicated that they would delay treatment if it was not available locally.

The case for implementing shared care services should be considered on a region by region basis taking into consideration factors such as patient caseload and regional demographic profile, including the requirements of culturally and linguistically diverse populations, Aboriginal people and an ageing population with hepatitis C, the capacity of GPs and physicians, and the availability of allied health services. In some regions, dedicated hepatitis C shared care services may inadvertently exacerbate the experiences of already stigmatised populations and a range of options should be made available.

Finally, GPs may have an increasing role to play in the future treatment landscape of hepatitis C if new oral-only treatments can be prescribed by GPs. It is timely to scrutinise and remove barriers to GP involvement in hepatitis C shared care and further explore the role of primary care in improving hepatitis C treatment uptake.

## Footnotes

1 The term ‘Aboriginal’ is used in preference to ‘Aboriginal and Torres Strait Islander’, in recognition that Aboriginal people are the original inhabitants of WA. No disrespect is intended to our Torres Strait Islander colleagues and community.

2 All states and territories in Australia offer a Patient Assisted Travel Scheme (PATS) which provides financial support for travel and accommodation costs to assist patients in rural and remote areas to access specialised health services if they are not available in their area. More information about the PATS in Western Australia is available at http://healthywa.wa.gov.au/Healthy-WA/Articles/N_R/Patient-Assisted-Travel-Scheme-PATS.
